# Recent Advances in Application of Polyoxometalates in Lignocellulose Pretreatment and Transformation

**DOI:** 10.3390/polym15102401

**Published:** 2023-05-22

**Authors:** Haoyu Deng, Wenbiao Xu, Dan Zhang, Xiangyu Li, Junyou Shi

**Affiliations:** 1 Key Laboratory of Wooden Materials Science and Engineering of Jilin Province, Beihua University, Binjiang East Road, Jilin 132013, China; dhy20421@163.com; 2 Key Laboratory of Biomass Materials Science and Technology of Jilin Province, Beihua University, Binjiang East Road, Jilin 132013, China; dzhang10482844461@163.com; 3 Collaborative Innovation Center of Forest Biomass Green Manufacturing of Jilin Province, Beihua University, Binjiang East Road, Jilin 132013, China; lixyv@126.com

**Keywords:** polyoxometalates, lignocellulose, pretreatment, transformation, biobased chemicals

## Abstract

Lignocellulose, composed of cellulose, hemicellulose, and lignin, holds immense promise as a renewable resource for the production of sustainable chemicals and fuels. Unlocking the full potential of lignocellulose requires efficient pretreatment strategies. In this comprehensive review, efforts were taken to survey the latest developments in polyoxometalates (POMs)-assisted pretreatment and conversion of lignocellulosic biomass. An outstanding finding highlighted in this review is that the deformation of the cellulose structure from I to II accompanied by the removal of xylan/lignin through the synergistic effect of ionic liquids (ILs) and POMs resulted in a significant increase in glucose yield and improved cellulose digestibility. Furthermore, successful integration of POMs with deep eutectic solvents (DES) or γ-valerolactone/water (GVL/water) systems has demonstrated efficient lignin removal, opening avenues for advanced biomass utilization. This review not only presents the key findings and novel approaches in POMs-based pretreatment but also addresses the current challenges and prospects for large-scale industrial implementation. By offering a comprehensive assessment of the progress in this field, this review serves as a valuable resource for researchers and industry professionals aiming to harness the potential of lignocellulosic biomass for sustainable chemical and fuel production.

## 1. Introduction

In the face of significant climate change and the depletion of fossil fuel reserves, the search for sustainable and renewable sources of energy has gained considerable momentum. Notably, lignocellulosic biomass has emerged as a promising alternative to conventional fossil fuels in recent years, garnering widespread recognition [1,2,3]. While fossil fuels are known for their non-renewability and adverse impact on the environment, lignocellulosic biomass derived from forest residues (e.g., wood, branches, leaves), agroforestry residues (e.g., corn stover, rice straw), and energy crops (e.g., switchgrass, boxwood) has emerged as a viable and environmentally sound alternative. This is due to their wide availability, abundance, affordability, and renewable nature, such as the contribution of biomass research to the mitigation of the greenhouse effect and global warming [4], to the achievement of sustainable development goals [5], and to the encouragement of circular economies [6], which also makes them important contributors to the biofuel sector [7,8,9]. The main challenge for the sustainable production of fuels and chemical intermediates from renewable biomass resources is the development of cost-effective processes for the conversion of highly functionalized feedstocks into value-added chemicals [10,11,12]. The capability of biomass to achieve a closed carbon cycle through the rapid regeneration of raw material feedstocks is undoubtedly of great benefit to human society. However, the awareness of these waste treasures is still very shallow, and a significant amount of biomass feedstock is usually burned or discarded, which is not only a waste of resources but is also a major contributor to the pollution of the atmosphere [13].

Lignocellulose is considered a promising feedstock for the production of biofuels, which are comparatively better and economical than other fuels [14,15], and they can be converted into platform chemicals [16,17], biopolymer [18,19], cosmetics [20], food additives [21], and other value-added products due to its abundance, renewability, and low cost [22,23]. It has been proved that the cellulose fibers are covered with hemicellulose, whereas lignin fills the spaces between the cellulose fibers [24,25]. However, many natural factors defined as biomass recalcitrance hinder the effective conversion of biomass, for instance, complex cell wall constituents, degree of lignification, cellulose crystallinity, and structural heterogeneity [26]. The presence of various obstacles suggests that the pretreatment of lignocellulose is a crucial stage in the conversion process. The implementation of efficacious pretreatment methodologies can facilitate the disintegration of the linkages between lignin and cellulose and hemicellulose, curtail cellulose crystallinity, enhance the surface area of the feedstock, augment enzyme accessibility to the substrate, and ultimately bolster the efficiency of enzymatic hydrolysis of cellulose [27,28]. At present, lignocellulosic pretreatment methods mainly include traditional physical methods (mechanical extrusion [29,30], ultrasonic [31], steam explosion [32,33], biochemical methods (dilute acid [34,35], dilute alkali [36], etc.), high temperature liquid water method [37], organosolv pretreatment [38], ILs pretreatment [39,40], etc. The high energy consumption and low lignin removal rate of mechanical extrusion make it the first step in the pretreatment process. It is recommended that mechanical extrusion be used in combination with other pretreatment methods to maximize its effect. Microwaves can disrupt the ultrastructure of lignocellulose, improve the sensitivity of enzymes, and have a good effect on the depolymerization of biomass. Nevertheless, the high energy consumption, the single reaction equipment, and the high cost of microwaves greatly limit its large-scale industrial application. Among these pretreatments, high temperature liquid water and steam explosion have a scant effect on the removal of lignin, which is a barrier to enzymatic hydrolysis [41]. The dilute acid alkali pretreatment could lead to the removal of hemicellulose lignin and increase the cellulosic yield in pretreated solid [42]. Thus, this could lead to enzymatic saccharification. However, for toxins generation, high metallurgy, chemical, and energy inputs are major barriers in commercial application [43]. Numerous approaches have been developed for the fractionation of lignocellulosic biomass. However, a majority of these methods necessitate the usage of costly chemicals such as ILs or involve the utilization of corrosive and hazardous compounds such as concentrated acids [44]. In general, there is no single biomass pretreatment method that can be economical, environmentally friendly, and efficient at the same time. Scientists are therefore investigating the design of integrated pretreatment and conversion processes to reduce the number of process steps and increase the yield of final products. The ultimate goal is to create sustainable and efficient processes that can convert lignocellulosic biomass into value-added products while minimizing environmental impact and improving economic viability for large-scale industrial applications [45,46].

POMs are a group of oxygenated polyacids, consisting of heteroatoms (P, Si, Fe, C, etc.) and polyatoms (M, W, V, Nb, Ta, etc.) bridged to metal atoms in a certain structure. According to their anionic structure, POMs can be mainly classified into Keggin [47], Anderson [48], and Dawson [49] types. The well-known compounds of POMs are phosphotungstic acid (H_3_PW_12_O_40_, PTA), silicotungstic acid (H_4_SiW_12_O_40_, STA), phosphomolybdic acid (H_3_PMo_12_O_40_, PMA), and silicomolybdic acid (H_4_SiMo_12_O_40_, SMA) [50]. POMs exhibit exceptional catalytic properties, serving as potent acid and redox catalysts. POMs consist of transition metal ions that are highly oxidized and linked to oxygen within a metal oxygen cluster. These solid acids are characterized by their versatility, possessing numerous absorption sites and robust redox properties that allow for precise control and manipulation of their acid and redox characteristics at the molecular level. As such, they offer tremendous potential in the optimization and design of acid and redox properties tailored to specific requirements in biomass conversion processes [51,52]. Due to their unique properties such as electron transfer and storage capacity, thermal stability, solubility in polar solvents, and structural flexibility, POMs are now widely used in medical, environmental, energy conversion, energy storage, catalysis, and other relevant fields [53]. POMs have been widely used as catalysts for homogeneous and heterogeneous acid-catalyzed reactions [54]. Some POMs are also strong oxidizing agents. When used for biomass hydrolysis, POMs show some progressive properties. For example, POMs exhibit exceptional acidity, even stronger than H_2_SO_4_, providing good acid sites for the hydrolysis of cellulose and hemicellulose, and have oxidative capacity so that they can simultaneously remove lignin by oxidative delignification, thus contributing to the hydrolytic exposure of cellulose. Therefore, POMs have been intensively used for the pretreatment of lignocelluloses to achieve high yields of the target products. However, POMs also have certain shortcomings, such as self-aggregation, which leads to shielding of the catalytic active site and thus lowers the catalytic efficiency. Further investigation is required to understand the underlying mechanisms, optimize the pretreatment conditions, and evaluate the impact on biomass composition and enzymatic digestibility.

Following the COVID-19 outbreak, there has been a noticeable escalation in the price of conventional fossil fuels. In contrast, the demand for biomass fuels has risen significantly since the pandemic has subsided, and it is anticipated that this trend will continue in the upcoming years. The future direction of biomass fuels is highly dependent on the development of efficient catalytic processes and the utilization of superior catalysts. The goal of this review is to find suitable, economical, and efficient biomass degradation systems so that it focuses on the advancements in the pretreatment of lignocellulose in various systems, such as GVL/water system, DES system, IL system, and other systems, utilizing POMs catalysts as the fundamental component. Additionally, this review discusses the complete conversion of lignocellulose catalyzed by POMs.

## 2. Advances in the Pretreatment of Lignocellulose with POMs in the GVL/Water System

The GVL/water system is an emerging pretreatment process for efficient fractionation of biomass [55,56,57]. With a low melting point (−31 °C), low vapor pressure, and good inter-solubility with water, GVL has received a lot of attention in recent years as a platform compound in the field of biorefining [58]. The employment of GVL as a pretreatment technique is held in high regard due to its intrinsic safety, recyclability, and renewable source as a solvent derived from the degradation and conversion of biomass. GVL-based pretreatment holds potential for closed-loop biomass conversion because GVL is a renewable liquid solvent derived from lignocellulosic biomass that can be easily separated and recycled efficiently by NaCl or liquid CO_2_, as evidenced by prior research [59]. Additionally, the solvent’s propensity for effective lignin solubilization in aqueous mixtures is a critical aspect of its utility [60]. Shuai [61] found that about 80% of poplar lignin was solubilized in the pretreatment solution after pretreatment at GVL/water ratio of 4:1, 120 °C, and 75 mM H_2_SO_4_ for 1 h. However, the cellulose conversion after GVL/water pretreatment was not as high as expected, and it was speculated that the esterification reaction on the cellulose surface might have occurred during the pretreatment process. His experiments were performed in GVL/water solvent system using 75 mM H_2_SO_4_ at 120 °C for mild biomass pretreatment. When GVL/water was used as a solvent, an increase in lignin removal from hardwoods was observed compared to other solvents, including ethanol, tetrahydrofuran, water (all containing dilute acids) and dilute base controls. The differences in glucose yields obtained during enzymatic digestion were even more pronounced between these solvents, and the use of GVL resulted in glucose yields of about 100%, which was three times higher than with tetrahydrofuran or ethanol and twenty times higher than with pure water. Sener [62] attempted to produce high yields of furfural from xylose and lignocellulosic biomass in a solvent system consisting of gentian violet (80 wt.%) and water (20 wt.%). A kinetic model of the xylose dehydration reaction was developed, including three reactions: dehydration of xylose to furfural, catalytic degradation of furfural, and catalytic degradation of xylose. The optimal temperature range for the dehydration of xylose to furfural in the GVL/water solvent system determined by the kinetic model is 480--500 K. Above a certain temperature (e.g., 473 K), higher yields of furfural (>90%) can be obtained.

Extensive experiments have been conducted to demonstrate that GVL pretreatment is a promising solvent for lignocellulosic biorefining. Raymond et al. [63] optimized conditions for the production of high cellulosic biomass and bioethanol from GVL pretreatment of Australian eucalyptus wood chips. The pretreatment parameters studied included GVL concentrations of 35–50% *w*/*w*, temperatures of 120–180 °C, and reaction times of 0.5–2.0 h. The optimal conditions were determined using response surface methodology (RSM) and central composite surface center design. The results showed that the concentration of GVL at 42.5–50% resulted in efficient separation of cellulose from eucalyptus wood chips for bioethanol production. After pretreatment with 50% GVL at 156 °C for 0.5 h, 39.9% of cellulose in untreated samples could reach 89.3% *w*/*w*. The bioethanol yield of GVL-treated eucalyptus under pre-hydrolysis with simultaneous saccharification and fermentation (PSSF) reached 94% of the theoretical yield. These results suggest that GVL is a feasible method for biological treatment of eucalyptus woodchips, and further research and development of GVL pretreatment is promising. 

**Figure 1:**
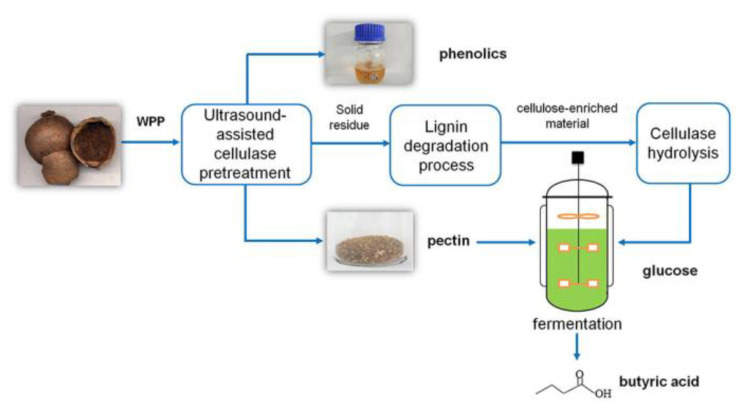
WPP’s high value utilization pathway. Adapted with permission from Ref. [64].

Pomegranate peel is an agricultural waste rich in carbohydrates and bioactive substances. Attempts have been made to efficiently convert waste pomegranate peels (WPP) into high value-added products (the process is shown in Figure 1). Wang et al. [64] first obtained a high level of phenolic compounds (12.2%) and bioactive pectin (24.8%) by enzymatic pretreatment. An integrated approach to lignin degradation using PTA, PMA, and STA as catalyst, biomass-derived GVL as sustainable solvent, and cellulose-catalyzed hydrolysis, respectively, was used. Characterization analysis revealed that the optimal degradation conditions were a temperature of 293 K, a reaction time of 3 h, and concentrations of 30, 40, and 30 mM for PTA, PMA, and STA, respectively. Under these conditions, the enzymatic efficiency was significantly increased, and the glucose yield of the cellulose feedstock obtained was 93.3%. Specifically, a practical system with POMs in GVL/water was used for the removal of lignin from the post-extraction WPP residue to obtain a cellulose-rich substrate material for further enzymatic degradation. Characterization of the substrate before and after degradation showed that the cellulose structure was not destroyed in the post-extraction WPP residue except for the removal of lignin under optimized pretreatment conditions. Importantly, the final WPP hydrolysate was not inhibited and yielded almost the same as butyric acid and glucose alone. This study provides an attractive process for the efficient use of biomass waste with good prospects for industrialization.

More and more attempts have been made in recent years to pretreat lignin with POMs in GVL/water instead of conventional H_2_SO_4_. Zhang et al. [65] obtained the optimal degradation conditions for lignin (130 °C, 3 h, 20 mM STA) by optimizing the catalyst concentration, the concentration of POMs, the reaction time, and the temperature. The cellulose-enriched materials were characterized by Scanning Electron Microscope (SEM) and Fourier Transform Infrared (FTIR), which showed that STA had efficient lignin degradation (97% removal of lignin was achieved) in GVL/water solvent with negligible damage to the cellulose in the logs. In addition, detailed enzymatic studies of the resulting cellulosic feedstock revealed its enzymatic applicability and great potential for future biorefinery applications as a starting material for the production of fermentable sugars from biomass. 

Due to the different structural and chemical properties of cellulose, hemicellulose, and lignin, an effective fractionation strategy is essential to separate each component without reducing the value of any component. Zhai et al. [66] proposed a lignocellulosic biorefinery method to fractionate poplar biomass into its individual components. Poplar wood was first pretreated in a CO_2_/H_2_O system to extract hemicellulose. About 87.90% of hemicellulose can be removed from the virgin feedstock in 10 min at 180 °C and 2 MPa CO_2_. Next, selective dissolution of lignin from the pretreated samples was carried out in GVL/water co-solvent and acid catalyst. It was found that STA exhibited higher catalytic delignification ability among the four catalysts (sulfuric acid, STA, PTA, and PMA). Under mild conditions (140 °C and 30 mM STA catalyst for 2 h), more than 91.35% of the original lignin was removed from the pretreated samples, and a high value of 90.76% cellulose purity was achieved in the cellulose-rich substrate (cellulose pulp). Overall, the present strategy has simple and green conditions and can provide an alternative option for efficient fractionation of lignocellulosic biomass. 

However, there are also many bottlenecks in current GVL research, and any GVL/water or GVL/solvent technology design must be able to demonstrate the cost-reducing economics of fuel and chemical production. In addition, the recycling of GVL is still in its infancy and may be an attractive research direction for GVL-based technology development. Existing methods are demanding in terms of equipment requirements and there is an urgent need to find efficient and convenient alternatives. GVL/water pretreatment requires further research to improve digestibility of pretreated substrates, reduce chemical requirements, and low toxin formation and high sugar release during enzymatic saccharification for renewable fuel and chemical production, which is currently the main bottleneck.

## 3. Advances in the Pretreatment of Lignocellulose with POMs in the DES System

DES are composed of a hydrogen bond acceptor (HBA) and a hydrogen bond donor (HBD) in different molar ratios [67,68]. Compared to conventional solvents and ILs, DES overcome many disadvantages and offer many advantages such as easy synthesis at mild temperatures and atmospheric pressure, no purification and waste disposal steps, regeneration, wide availability, biocompatibility, and low cost. A new generation of green solvents, DES, has received a lot of attention in recent years as DES can effectively reduce the recalcitrant nature of the biomass that results in an increase in the sugar and product yields after the enzymatic saccharification and fermentation process and has shown excellent performance in the field of pretreatment [69]. 

To improve the pretreatment performance of neutral DES (choline chloride/glycerol), three environmentally-friendly POMs (PTA, PMA, and STA) are usually used as catalysts. Guo et al. [70] experimentally found that STA pretreated at 120 °C for 3 h gave 97.3% enzyme digestibility when the enzyme loading was 15 FPU/g substrate, which was about eight times higher than that of the raw material sample. More importantly, 80% glucose yield could be obtained within 12 h. Meanwhile, an ethanol yield of 81.8% was obtained in the semi-simultaneous saccharification and fermentation (SSSF) process. The efficient conversion was attributed to a significant delignification (89.5%), which resulted in exposing a more accessible specific surface area. Overall, this work provides a very efficient and environmentally-friendly method for converting biomass to biofuels under mild conditions. Guo et al. [71] also found that neutral DES pretreatment was not effective in the pretreatment of Miscanthus × giganteus (the removal of lignin was only about 4.4%). Therefore, a neutral DES (choline chloride/propanetriol) catalyzed by environmentally-friendly POMs (PTA, PMA, and STA) was used for pretreatment. The treated Miscanthus × giganteus had an exfoliated, rough, and porous surface with a slight increase in crystallinity. The enzymatic conversion was as high as 80% in a relatively short enzymatic time (12 h) and its conversion was 97.3% after 72 h of enzymatic hydrolysis. The ethanol yield increased from 9.9% to 81.8% using a SSSF process, which is about eight times higher than the ethanol yield of untreated Miscanthus × giganteus. Using the same system described above, Guo prepared lignin nanoparticles with excellent antioxidant properties using a POMs-catalyzed neutral DES in a clean and efficient manner. The chemical structural changes of lignin were elucidated by Gel Permeation Chromatography (GPC), SEM, Transmission Electron Microscope (TEM), and FTIR. It was found that the STA-catalyzed choline chloride/propanetriol treatment of Miscanthus × giganteus at 120 °C for 3 h resulted in a lignin yield of 78.2%. This was attributed to STA as a quaternary acid providing more protons to selectively break the ether bond and further facilitate the removal of lignin. 

PTA-assisted DES has noteworthy synergistic effects in breaking lignin–carbohydrate complex (LCC) bonds and decomposing corn stover. Xie et al. [72] chose an environmentally-friendly POMs, PTA, as a catalyst to assist the decomposition of corn stover with a neutral DES. The reaction was carried out at 110–150 °C for a short period of time. The results obtained by chromatographic analysis and infrared spectroscopy showed that most of the lignin (86.1%) and hemicellulose (89.7%) were removed, while cellulose was obtained in high yields (88.9–94.3%). Benefiting from the removal of hemicellulose and lignin, the enzymatic digestibility of the pretreated material was significantly increased to 94.4%, which was 5.3 times higher than that of the untreated corn stover. Meanwhile, the regenerated lignin nanoparticles had a high purity (over 85%) and a low and uniform molecular weight (5669–2437 g/mol). In addition, PTA/DES exhibited excellent recycling performance. Overall, the technically feasible PTA-catalyzed DES pretreatment provides insights for future effective deconstruction of corn stover recalcitrance for crop valorization.

DES are efficient and inexpensive green solvents with similar physicochemical properties as ILs but with lower toxicity and cost, and better biocompatibility and biodegradability. Recently, DES have shown great potential for biomass pretreatment. Carboxylic acid-based DESs exhibit better delignification and better enzymatic conversion rates. However, long-term use of carboxylic acids may corrode equipment and cause environmental pollution. However, the toxicity and environmental pollution of DES still limit its large-scale application.

## 4. Advances in the Pretreatment of Lignocellulose with POMs in the ILs System

ILs are salts composed of organic cations and inorganic or organic anions that are liquid at room temperature or near room temperature [73]. ILs evolved from the traditional high-temperature molten salts, but have very different properties and behavior compared to the general ionic compounds. The biggest difference is that the general ionic compounds can only become liquid at high temperatures, while ILs are liquid in a wide range of temperatures near room temperature, with the lowest freezing point reaching −96 °C. Although ILs are composed of ions, their composition is variable, so they become “design solvents [74]”, and the most important advantage is that the vapor pressure is almost undetectable, unlike traditional industrial organic solvents, so they are also called “green solvents”, and the designability of ILs creates unlimited possibilities for their effects. Ionic liquids are well known for reducing the crystallinity of cellulose and changing the crystalline structure of cellulose to an amorphous state that is more easily digested by enzymatic hydrolysis [75]. The acceleration of enzymatic digestion of pretreated biomass is due to the higher hydrogen bonding alkalinity of the ILs, which facilitates delignification and hemicellulose removal, and the transition from cellulose I to cellulose II leads to a more amorphous nature [76].

**Figure 2:**
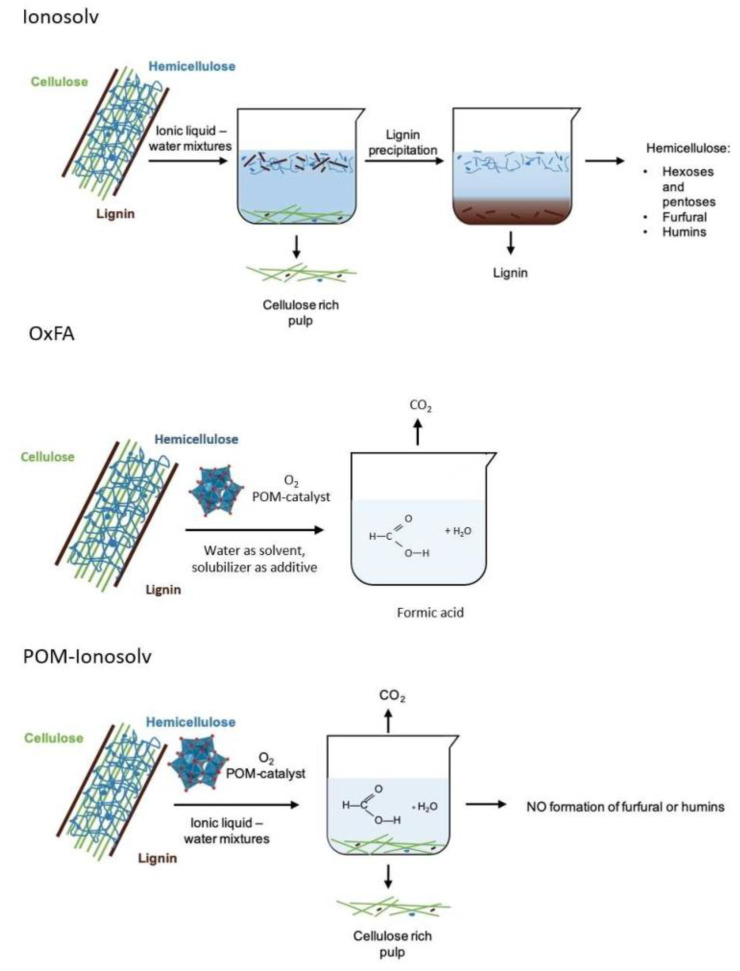
Conversion pathways of lignocellulosic substrate with Ionosolv pretreatment, in OxFA process in pure water, and POM–Ionosolv concept. Adapted with permission from Ref. [77].

Bukowski et al. [77] developed an innovative process for the separation of lignocellulosic biomass using low-cost ILs (Ionosolv) and the production of bio-derived formic acid using POMs and molecular oxygen (OxFA process) (the process is shown in Figure 2). The catalyst was characterized by FTIR, Gas Chromatography (GC), High Performance Liquid Chromatography, and Nuclear Magnetic Resonance techniques, confirming stability and stable formic acid yields over three consecutive POM-Ionosolv cycles. High formic acid yields of 26% (pine chips), 23% (beech chips), and 18% (manzanita) were obtained relative to the initial carbon content of the biomass, as well as an unprecedented formic acid oxidation selectivity of 54–62%, depending on vanadium substitution in the POMs, treatment temperature, and water content in the reaction mixture. The results showed that hemicellulose and some lignin were selectively dissolved in the ionic liquid hydrogen triethylamine sulphate and oxidized in site by the Keggin-type polymetallic POMs to short-chain distillable carboxylic acids in high yields and selectivity. With further optimization, this concept has the potential to generate two chemical products directly from lignocellulose in high yield and selectivity, thus becoming a new way to fully utilize cellulose, hemicellulose, and lignin.

It is well known that the designability of ILs is their greatest advantage. The oxidative decomposition of lignocellulose was carried out in a newly developed polymetallic ox imidazole ionic liquid mixture by Shi et al. [78]. Aromatic compounds, including acids, esters, ketones, aldehydes, and phenols, were selectively produced under different conditions. Gas Chromatograph–Mass Spectrometer (GC–MS) characterization of the distribution and residues of lignocellulosic decomposition products under different conditions showed that 19 phenolic compounds dominated with a reaction selectivity of 45.1% and a yield of 4.3%. Low temperatures and more oxidants favored the production of aromatic acids. Interactions between reactants led to reasonable acidity and oxidation of P_2_W_17_, which favored electron transfer and decomposition of lignocellulose. It was found that [Emim]HSO_4_ could be recycled five times. This work not only develops a new catalytic system but also provides guidance for product-oriented lignocellulosic decomposition. 

The lignin-first concept is a new innovation that fully utilizes lignocellulose for the production of high value-added chemicals. Ionic liquid (IL) polymetallic oxides (MIMPS)_2_H_4_P_2_Mo_18_O_62_ (MIMPS = 1-(3-sulfo) propyl-3-methylimidazole) were reported to be active for the cleavage of β-O-4, α-O-4, and 4-O-5 bonds in all three lignin models and were also effective for the conversion of natural lignocellulose. Li et al. [79] used a one-pot, three-step method for soft and hard lignocelluloses in a one-pot, three-step method to depolymerize the three components. Characterization showed that for soft lignocellulose (*Pinus sylvestris*), lignin was decomposed to guaiacol and phenol at 130 °C and 14 h with yields of 15.3% and 12.9%, respectively, with delignification efficiency of 98.6%. Meanwhile, hemicellulose and cellulose remained intact during delignification, and were hydrolyzed to 3.5% xylose at 150 °C and 14 h, and to 36.4% glucose at 170 °C and 12 h, with a delignification efficiency of 100%. For hard lignocellulose (poplar), the yield of guaiacol and phenol was 10.7% and 8.7%, respectively, at 130 °C, 14 h, with a delignification efficiency of 91.9%, while the hemicellulose conversion efficiency was 90.4% at 150 °C, 12 h. Glucose was 100% at 170 °C, 12 h, with a cellulose conversion efficiency of 32.9% for xylose. The experiment also found that [MIMPS]_2_H_4_P_2_Mo_18_ exhibited high efficiency and reusability in the one-pot, three-step efficient conversion of small molecules, as well as easy separation in ten cycles due to temperature reversibility. Therefore, controlling the reaction time and temperature of the three-step reaction is an important factor to achieve complete conversion of natural lignocellulose by [MIMPS]_2_H_4_P_2_Mo_18_. Today, ILs are receiving increasing attention for their better designability, which has led to a significant increase in the production of fermentable sugars and bioethanol. However, the high cost of preparation, economic sustainability, and toxicity hinders their industrialization.

## 5. Advances in the Pretreatment of Lignocellulose with POMs in Other Systems

In addition to the above popular pretreatment systems, there are also many biphasic systems with water such as n-butanol, cresol, etc., as well as the use of enzymatic catalysis, the addition of sulfone organic solvents, and other means. These methods have produced a good effect on the conversion of biomass. Hronec et al. [80] proposed an alternative method for the production of bio-based chemicals using terephthalic acid as a recoverable acid catalyst. Selective fractionation of lignocellulose-cellulose was carried out in a biphasic system containing water and n-butanol in the presence of a terephthalic acid catalyst. The products of fractionation were aqueous solutions of sugars (mainly oligosaccharides), solid cellulose slurry, and lignin dissolved in the n-butanol phase. The resulting hydrolysate was free of acidic catalysts and contained very low concentrations of furans and other inhibitors. About 50.9% of the sugars (as monomers and oligomers) in the hydrolysate was converted to microbial oils. The cellulose slurry containing precipitated terephthalic acid could be upgraded to formic and acetic acids by oxidation of phosphomolybdenum-vanadium POMs and favorably influenced the subsequent catalytic oxidation process; the yield of formic acid was increased by 10.6%. Terephthalic acid is precipitated almost quantitatively from the reaction solution at room temperature and can be recovered very simply and efficiently and directly into the fractionation step, which is very much in line with the concept of green chemistry. 

**Figure 3:**
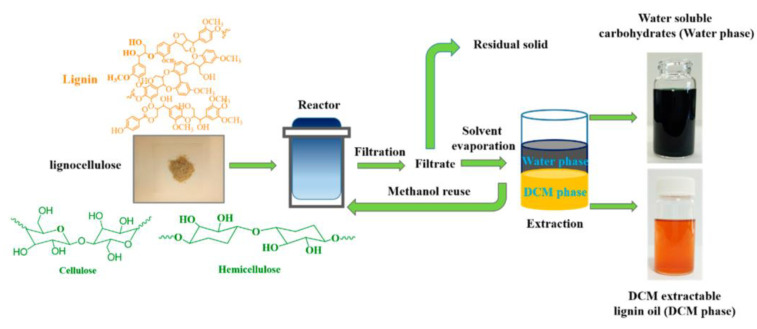
Schematic of the overall processes of the fractionation of lignocellulosic biomass. Adapted with permission from Ref. [81].

Effective methods for converting sustainable lignocellulosic biomass into high-value chemicals are of great importance for the utilization of biomass. Although many conversion methods have been proposed, those commonly used require demanding operating conditions. Yang et al. [81] designed a new process (shown in Figure 3) without using technical lignin but used an oxidation method with polymetallic oxides (H_3_PMo_12_O_40_) as catalysts for the direct conversion of natural lignocellulose in methanol/water mixtures to produce high-value chemicals. Characterization by GC–MS chromatography revealed that 80% of lignin in hardwoods was hydrolyzed at 150 °C and 1 MPa O_2_ for 2 h to give a low molecular weight lignin oil (376 Da). The aromatic hydrocarbon monomer yield of lignin oil was 38.0% by total lignin mass. Meanwhile, only 9.2% of solids remained; 83.7% of hemicellulose was converted to water-soluble carbohydrates. This process has achieved good lignin oil yields with simple operating conditions and is worthy of reference.

**Figure 4:**
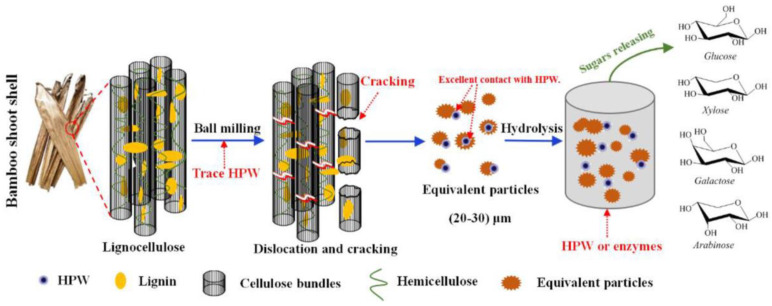
The integrative process for efficient saccharification of BSS–HPW into monosaccharides. Adapted with permission from Ref. [82].

The pristine carbohydrate–lignin matrix exhibits low reactivity due to the presence of crystalline bands and lignin inhibition. To overcome these barriers, Xiao et al. [82] proposed a synergistic combination of ball milling, POMs (PTA), and enzymatic processes (shown in Figure 4) to unlock the lignin cage to release sugars. In particular, the ball milling technique is an alternative pretreatment method for the recrystallisation of cellulose from bamboo shoot shells, resulting in a crystallinity index (CrI) of 71.3% for cellulose I and 9.5% for cellulose II, accompanied by a reduction in particle size from 283 μm to 29 μm (D50). The results showed that under optimal conditions, hydrolysis of PTA at 150 °C for 2.0 h followed by enzymatic digestion at 50 °C for 7.0 h yielded 763.6 mg/g monomeric sugar with a substrate of 0.7 g PTA/g for BSS without PTA milling, while 2.5% PTA milling yielded 741.6 mg/g monomeric sugar with a substrate of 0.1 g PTA/g for BSS. The results showed that ball milling had a large effect on the particle size and crystallinity of the lignocellulose, resulting in a significant loosening of the recalcitrant structure of the lignocellulose. This comprehensive strategy provides a good guide to promote the efficient conversion of lignocellulosic raw materials into monosaccharides.

Effective pretreatment to reduce the recalcitrance of lignocellulosic biomass is considered an important prerequisite for lignocellulosic valorization. Liu et al. [83] developed a POMs-assisted (i.e., PTA, STA, and PMA) pretreatment with sulfone organic solvent to reduce willow recalcitrance for effective saccharification cation and subsequent lignin valorization. The pretreatment significantly improved cellulose accessibility with a high glucose yield of 94.77%, a 13.3-fold increase compared to 7.05% in the original willow. In addition, the recovered lignin had high purity (∼98%), medium molecular weight (3378–3585 g/mol), low polydispersity (∼2.0), and good antioxidant properties (RSI value of 1.18). By isolating lignin, lignin nanospheres with different morphologies were prepared, which increases the potential applications of lignin. Overall, POMs-assisted silane pretreatment proved to be a promising method for cellulase hydrolysis and lignin valorization. 

**Figure 5:**
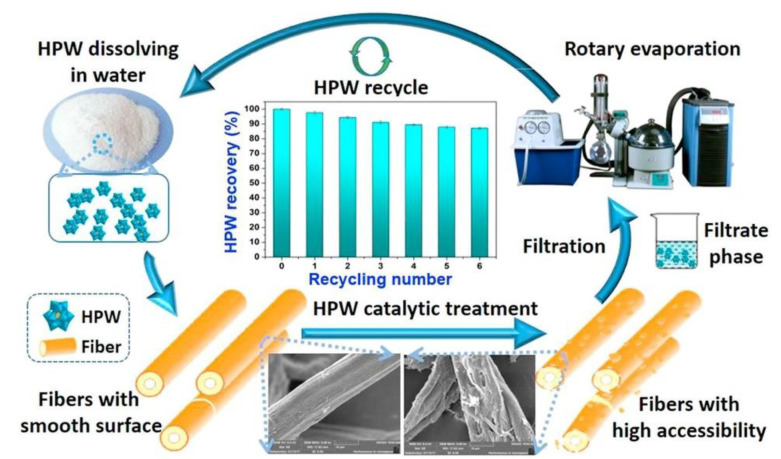
Schematic concept of enhancing the accessibility reactivity of dissolving pulp by using HPW catalytic treatment. Adapted with permission from Ref. [84].

In general, the mechanism of HPW hydrolysis of cellulose is similar to that of conventional inorganic acids such as dilute sulfuric and hydrochloric acids. However, HPW is expected to have good catalytic efficiency, probably because of the high Bronsted acidity of HPW and the formation of cellulose-HPW^3−^ intermediates from the POMs (HPW^3−^) produced during cellulose hydrolysis, thus reducing the apparent activation energy and thus improving the catalytic reaction efficiency. Wang et al. [84] attempted to improve the availability/reactivity of dissolving pulp with HPW catalytic treatment (conceptual diagram shown in Figure 5). The results showed that the total pore volume, average pore size, SSA, and WRV of the synthesized pulp increased from 1.93–2.56 mL/g, 6.92–8.04 nm, 1.43–2.96 m^2^/g, and 101–122%, respectively. As a result of these good fiber accessibility changes, the Fock reactivity of the obtained dissolving pulp increased from 49.1% to 74.1% and the viscosity decreased from 561 mL/g to 437 mL/g. The used HPW could be recovered, and the results showed that the recovered HPW maintained 87.1% of its catalytic activity after six cycles. It can be seen that the recovery of HPW has great potential for economic benefits and industrial applications. The HPW catalytic treatment concept offers a green option for the production of high-quality dissolving pulp.

## 6. Advances in the Complete Conversion of Lignocellulose Catalyzed by POMs

### 6.1. Reduction Conditions

Conversion of biomass under reducing conditions refers to the selective depolymerization of lignocellulose into chemicals such as fuels in the presence of catalysts, molecular hydrogen, and solvents. The catalysts referenced as Rux/Cuy@CsWP and Rux/Cuy@CsMoP are synthesized by wet impregnation of the ruthenium and/or copper precursors on the acidic supports Cs_2.5_H_0.5_PW_12_O_40_ (CsWP) and Cs_2.5_H_0.5_PMo_12_O_40_ (CsMo). Garron et al. [85] studied the direct catalytic thermostatic conversion of wood in the presence of molecular hydrogen. The catalyst consisted of Cs-exchanged POMs of the above two types, loaded with versatile Cu-Ru, and the organic liquids obtained included saturated alkanes and aromatics in yields up to 30%. Importantly, the oxygen content is about 3 wt.% (<5 wt.% required for biofuel formulations) and has a higher oxygen content and a higher calorific value of 41 MJ kg^−1^, which is very close to the standard diesel fuel used in motor fuels (44 MJ kg^−1^). This catalyst might be applicable for the valorization of wood waste such as lignin from black liquor or municipal solid waste. 

**Figure 6:**
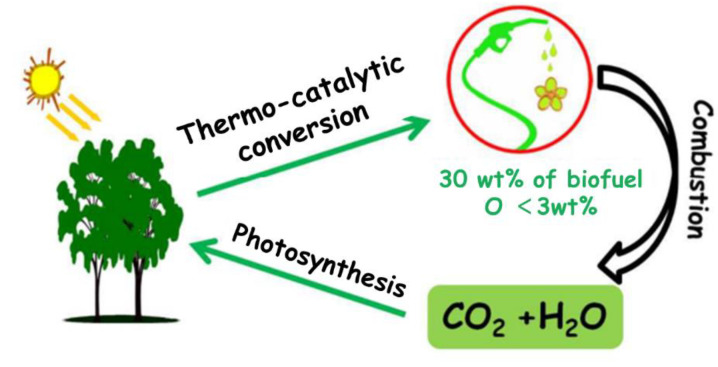
A well-dispersed Ru-Cu on POM catalyst converts pine wood into biofuel (O = 3.1 wt%) with a yield up to 30 wt.%. Adapted with permission from Ref. [86].

Maksoud et al. [86], from the same team as Garron, went on to investigate the catalytic activity of multifunctional catalysts for hydrogen-catalyzed conversion of pine wood to biofuels in a batch reactor (see Figure 6 for the process). Ruthenium and copper bimetallic catalysts (RuxCuy@CsPW) with PTA ions as carriers were also prepared by wet leaching of ruthenium and copper precursors using a strongly acidic carrier (CsPW). The synergistic effect induced the formation of smaller bimetallic nanoparticles compared to the single metal. In the presence of the catalyst, the separation of the organic liquid was 30%, while in the absence of the catalyst, only trace amounts of organic liquid were obtained. GC–MS analysis showed that the organic phase consisted of saturated alkanes and aromatics with an oxygen content of 3.1 wt.%. The higher heating value of the organic phase was 41 MJ kg^−1^ (diesel = 44 MJ kg^−1^). This work demonstrates the possibility of one-step catalysis for the conversion of biomass into biofuels.

### 6.2. Oxidation Conditions

The yield of lignin monomers during lignin depolymerization is known to be limited by the irreversible condensation of lignin during fractionation and/or depolymerization. Du et al. [87] proposed an oxidative catalytic fractionation depolymerization strategy for the efficient depolymerization of lignin using PMo_12_ as the sole catalyst and oxygen as the oxidant. The lignin (and lignin fragments) was separated from wood chips by lignin methoxylation with α-OH using a low (0.025 mmol) concentration of PMo_12_ catalyst in a mixture of methanol and water under mild conditions (100 °C). α-OH methoxylation of POM in methanol resulted in a high degree of delignification (96.2%), thus effectively avoiding lignin condensation during the lignin conversion. The isolated lignin was further oxidized to lignin-derived compounds by Gas Chromatography—Flame Ionization Detector spectroscopy in 74.0% yield, including 45.9% for the aromatic monomer. The remaining cellulose can be further used for the production of bioethanol, resulting in complete stabilization of the biomass. Finally, the methods described here will stimulate the development of novel biorefineries.

### 6.3. Other Conditions

Bioethanol has been widely accepted by scientists worldwide as an alternative to fossil fuels, and it is currently the focus of research in the field of biomass. Nayak et al. [88] investigated the feasibility of producing total reducing sugars (TRS) from five different biomass feedstocks, namely mangosteen, sugarcane leaves, willow jelly, sunflower seeds and bamboo leaves, through compositional analysis and catalytic biomass hydrolysis. Among the five biomasses tested, the highest TRS (5.77 wt./dry wt.%) was obtained for willow jelly at a HPMo catalyst to biomass ratio of 30:100 (wt./wt.%), and the reaction temperature was 120 °C for 3 h. However, a catalyst to biomass ratio of 20:100 (wt./wt.%) was economic (5.25 wt./dry wt.%). POMs is a green acid and its acidity can be changed according to the need. In the tests, HPMo was found to be the most efficient catalyst for the application (TRS production) with a yield of 5.77 wt./dry wt.% of willow jelly TRS. The selection of the optimal process parameters was also investigated. The optimal hydrolysis conditions were 20:100 ratio of HPMo to willow jelly (wt./wt.%), with a reaction time of 3 h and a temperature of 120 °C. In addition, willow jelly has an abundance of obtainable an additional advantage of the shortest growth period and cellulose percentage. Therefore, willow jelly and HPMo are recommended as the preferred biomass and solid acid catalysts for high yield production of TRS.

**Figure 7:**
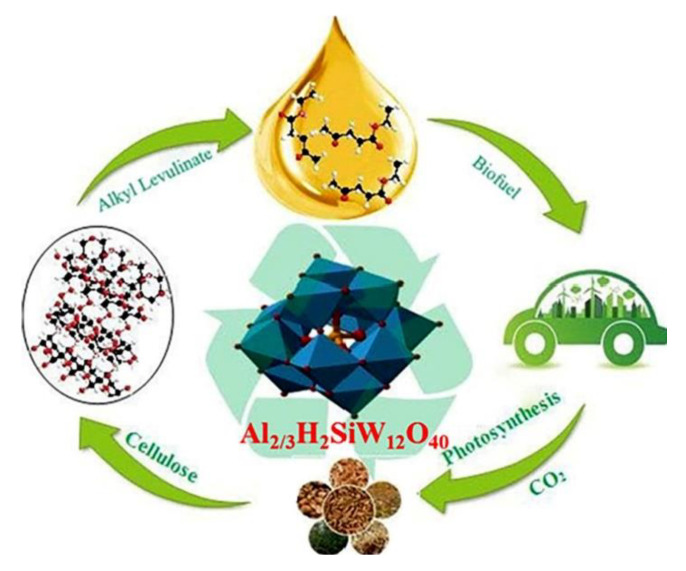
Schematic diagram of stabilization of cellulose and lignocellulosic biomass to alkyl levulinate. Adapted with permission from Ref. [89].

Tao et al. [89] designed metal-modified POMs with tunable Lewis and Brønsted acid sites as monolithic multifunctional catalysts to valorize lignocellulosic biomass to alkyl levulinic (AL) by a single process under mild conditions (as shown in Figure 7). Therefore, high yields of AL were obtained, where Al-exchanged two protons of H_4_SiW_12_O_40_ (Al_2/3_H_2_SiW_12_O_40_) was found to be uniquely effective. H_4_SiW_12_O_40_ is marked as HSiW and Al_2/3_H_2_SiW_12_O_40_ is marked as AlHSiW in the following. The apparent activation energy was calculated by Arrhenius’ law. Kinetic and mechanistic studies showed that the introduction of Al^3+^ significantly promoted cellulose depolymerization and glucose–fructose isomerization, allowing the glycosidic linkages of cellulose polymers to break more easily and the reactions to proceed under milder operating conditions. The activation energies for cellulose degradation and glycocalyx decomposition decreased from 186.3 kJ/mol and 129.2 kJ/mol to 135.7 kJ/mol and 92.9 kJ/mol, respectively. Thus, the direct use of cellulose over AlHSiW greatly improved the reaction rate and improved the selectivity of AL. The prepared catalyst has good flexibility for lignocellulosic feedstock and linear alcohol solvent with 50–72% yield of AL. The prepared catalyst, solvent, and product can be separated from the reaction mixture and the recovered catalyst has good reusability and stability. This strategy offers many advantages over the production of biomass-based AL, such as direct use of cellulosic materials, avoidance of expensive or dual catalysts, high efficiency, and environmental friendliness. This study opens a reliable and promising pathway for the one-pot conversion of three major components of lignocellulose to esters.

**Figure 8:**
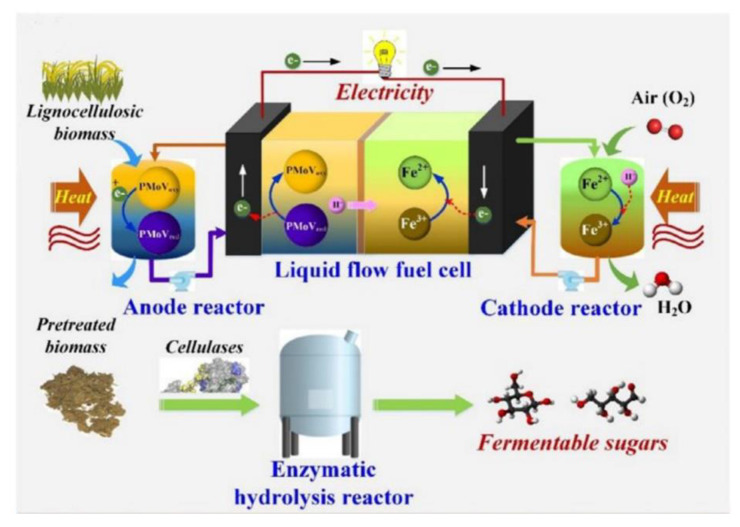
Schematic diagrams of the coupled process of biomass pretreatment with direct biomass-to-electricity conversion with molybdovanadophosphoric acid and ferric ion as electron carriers. The working principle of the coupled process. Adapted with permission from Ref. [90].

Due to their acidity, oxidative capacity, and redox reversibility, molybdovanadophosphoric POMs (H_n+3_PMo_12−n_V_n_O_40_, abbreviated as PMo_12−n_V_n_) were used as electron transfer carriers for coupling biomass pretreatment enzymatic hydrolysis and the direct conversion of biomass into electrical energy (schematically shown in Figure 8). In this new coupling process, PMo_12−n_V_n_ pretreatment led to the deconstruction of the cell wall structure while PMo_12−n_V_n_ was reduced, which can be considered as a “charging” process. The reduced PMo_12−n_V_n_ was further re-oxidized in a liquid flow fuel cell (LFFC) and released electrons to generate electricity, which is the “discharge” process. Yang et al. [90] prepared several Keggin-type PMo_12−n_V_n_ with different degrees of vanadium substitution (DS_V_). Compared to Keggin-type PMA (PMo_12_), PMo_12−n_V_n_ (n = The enzymatic digestibility of cellulose in PMo_12−n_V_n_) pretreated wheat straw generally decreased with increasing DS_V_, but the enzymatic digestibility of lignin generally increased with increasing DS_V_. The highest enzymatic glucan conversion was obtained for PMo_12_-pretreated wheat straw at 120 °C, reaching 95%, followed by PMo_11_V_1_ pretreatment (85%). Discharging reduced heteropolysaccharides in the LFFC showed that vanadium substitution increased the maximum output power density (Pmax). When using FeCl_3_ as the cathode electron carrier, PMo_9_V_3_ obtained the highest Pmax (44.7 mW/cm^2^), while PMo_12_ obtained the lowest Pmax (27.4 mW/cm^2^). All POMs showed good electrode Faraday efficiencies (>95%) and cell discharge efficiencies (>93%). Based on the calorific value of the product and the electrical energy generated, the energy efficiency of the coupling process ranged from 18–25%, depending on the DS_V_. PMo_12_ and PMo_11_V_1_ appeared to be the most suitable POMs for mediating the coupling process.

## 7. Conclusions

Through advanced spectroscopic techniques and computational modeling, researchers have gained valuable insights into the interactions between POMs and lignocellulosic biomass. The use of GVL/water, DES, and ILs as solvents in combination with POM catalysts has demonstrated enhanced lignocellulose dissolution, improved sugar release, and reduced formation of inhibitory byproducts. Additionally, POMs exhibit unique physical and chemical properties that make them suitable for use in both homogeneous and non-homogeneous systems, and their recyclability with minimal loss of catalytic activity is another benefit. These POMs-based systems offer greener alternatives to traditional harsh pretreatment methods, providing opportunities for more sustainable and efficient biomass valorization. However, the unclear mechanism of POMs action may somewhat limit its large-scale application, which remains to be identified through further research by scientists. However, it is undeniable that, in the future, POMs catalysts are expected to be used in a variety of emerging and efficient catalytic systems to make the utilization of biomass more economical and efficient. As an emerging market, the demand for POMs catalysts is expected to increase rapidly in response to the requirements of biomass conversion for green economic development. In summary, this review article highlights the potential of emerging POMs-based catalytic systems for biomass pretreatment and conversion and provides suggestions for improvement.

## Data Availability

Not applicable.
